# Supplementary material for: AP-2δ Is the Most Relevant Target of AP-2 Family-Focused Cancer Therapy and Affects Genome Organization
Source: Cells. 2022 Dec 19;11(24):4124. doi: 10.3390/cells11244124 (PMC9776946; doi:10.3390/cells11244124)
Supplement: Supplementary file 1 [file cells-11-04124-s001.zip › Table_S1.pdf]

Table S1. Pairwise comparisons between AP-2 factors with additional all-by-all percentage of identity.

Comparison	Percentage of identity	Included in Figure 2A?
TFAP2A vs TFAP2B	66.026%	
TFAP2A vs TFAP2C	60.965%	
TFAP2A vs TFAP2D	45.188%	✓
TFAP2A vs TFAP2E	57.456%	
TFAP2B vs TFAP2C	56.144%	
TFAP2B vs TFAP2D	43.340%	✓
TFAP2B vs TFAP2E	58.071%	
TFAP2C vs TFAP2D	41.365%	✓
TFAP2C vs TFAP2E	51.073%	
TFAP2D vs TFAP2E	42.505%	✓
All-by-all comparison	29.377%	✓
