# Supplementary figures and images for: AP-2δ Is the Most Relevant Target of AP-2 Family-Focused Cancer Therapy and Affects Genome Organization
Source: Cells. 2022 Dec 19;11(24):4124. doi: 10.3390/cells11244124 (PMC9776946; doi:10.3390/cells11244124)

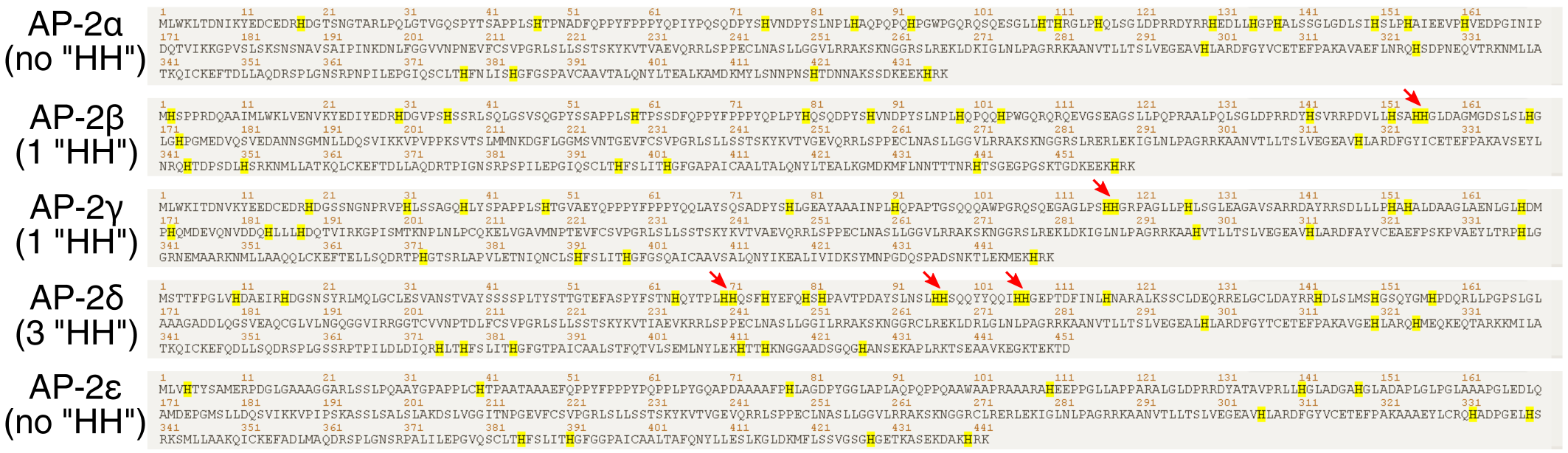

Supplement: Supplementary file 1 [file cells-11-04124-s001.zip › Figure_S1.tif]

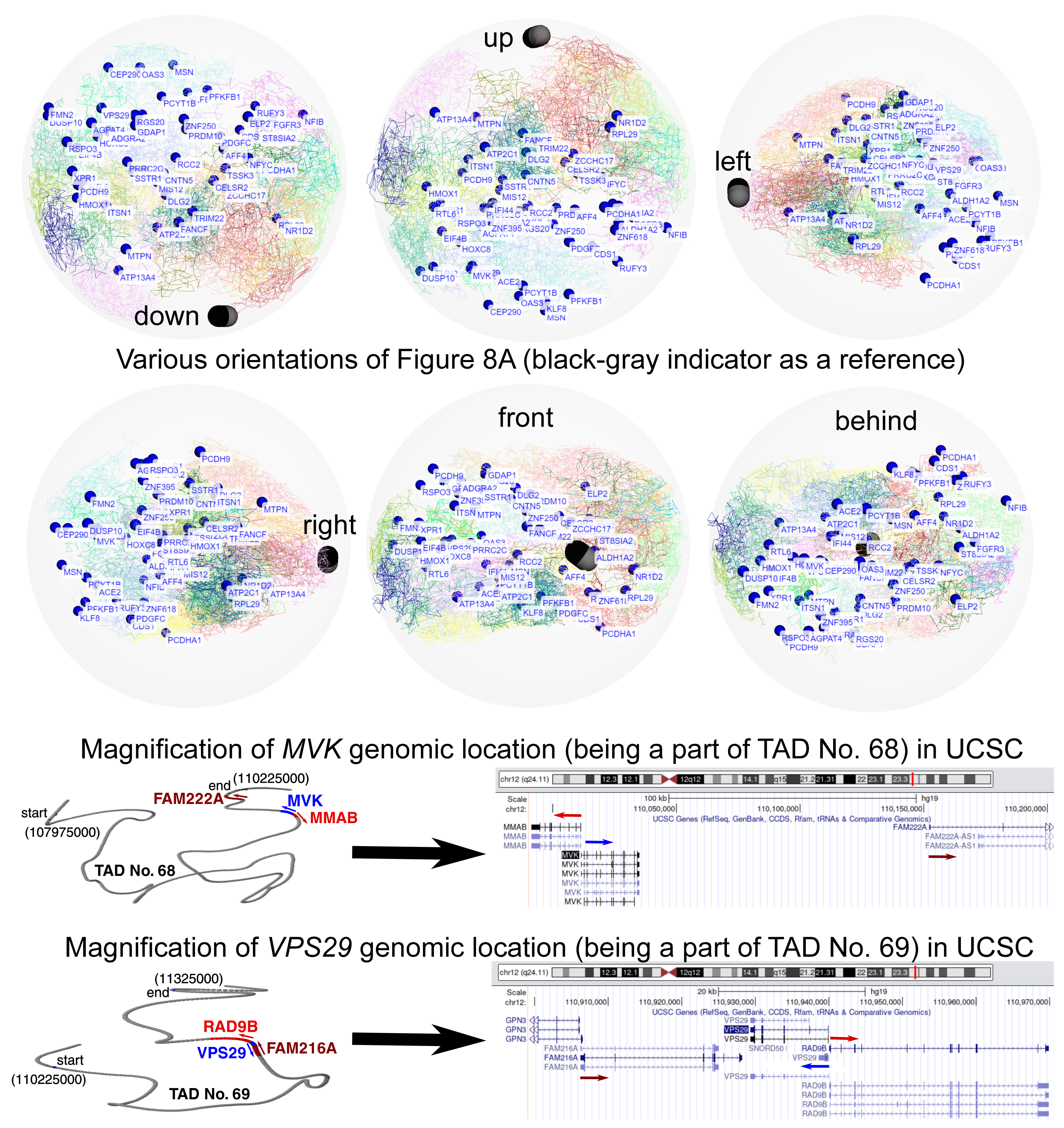

Supplement: Supplementary file 1 [file cells-11-04124-s001.zip › Figure_S2.tif]
